# Investigation of methicillin-resistant *Staphylococcus aureus* among clinical isolates from humans and animals by culture methods and multiplex PCR

**DOI:** 10.1186/s12917-018-1611-0

**Published:** 2018-10-03

**Authors:** M. M. Rahman, K. B. Amin, S. M. M. Rahman, A. Khair, M. Rahman, A. Hossain, A. K. M. A. Rahman, M. S. Parvez, N. Miura, M. M. Alam

**Affiliations:** 1 Department of Medicine, Faculty of Veterinary Science, Bangladesh Agricultural University, Mymensingh, 2202 Bangladesh; 2 Department of Microbiology & Hygiene, Faculty of Veterinary Science, Bangladesh Agricultural University, Mymensingh, 2202 Bangladesh; 3 Department of Microbiology, Mymensingh Medical College, Mymensingh, Bangladesh; 4 Veterinary Teaching Hospital, Joint Faculty of Veterinary Medicine, Kagoshima University, Kagoshima, Japan

**Keywords:** *Staphylococcus aureus*, MRSA, Animals, Humans, Bangladesh

## Abstract

**Background:**

*Staphylococcus aureus* is responsible for large numbers of hospital-related and community-acquired infections. In this study, we investigated the presence of *S. aureus* and methicillin-resistant *S. aureus* (MRSA) in 100 samples from animals (55 cattle, 36 dogs, and 9 cats) and 150 samples from hospitalized human patients. The samples were collected from healthy and diseased animals and from diseased humans and included milk, wound swab, pus, exudates, nasal swab and diabetic ulcer. Initially, *S. aureus* was isolated and identified by colony morphology, Gram staining, and biochemical tests (catalase and coagulase tests). The *S. aureus*-positive samples were examined by polymerase chain reaction (PCR) to determine their MRSA status.

**Results:**

Of the 100 animal samples, 29 were positive for *S. aureus*. Four samples (13.8%) from dogs were MRSA-positive, but samples from cattle and cats were MRSA-negative. Of the 150 human samples we collected, 64 were *S. aureus*-positive and, of these, 34 (53.1%) were MRSA-positive. Most (28%) of the MRSA samples were isolated from surgical wound swabs, followed by the pus from skin infections (11%), exudates from diabetic ulcers (6%), exudates from burns (4%), and aural swabs (3%). By contrast, a low MRSA detection rate (*n* = 4) was seen in the non-human isolates, where all MRSA bacteria were isolated from nasal swabs from dogs. The antimicrobials susceptibility testing results showed that *S. aureus* isolates with *mec*A genes showed resistance to penicillin (100%), oxacillin (100%), erythromycin (73.5%), ciprofloxacin (70.6%), and gentamicin (67.7%). The lowest resistance was found against ceftazidime, and no vancomycin-resistant isolates were obtained.

**Conclusions:**

We detected *S. aureus* and MRSA in both human and canine specimens. Isolates were found to be resistant to some of the antimicrobials available locally. MRSA carriage in humans and animals appears to be a great threat to effective antimicrobials treatment. The prudent use of antimicrobials will reduce the antimicrobial resistance. Our findings will help to find the most appropriate treatment and to reduce antimicrobial resistance in the future by implementing prudent use of antimicrobials. Further studies are required to better understand the epidemiology of MRSA human–animal inter-species transmission in Bangladesh.

**Electronic supplementary material:**

The online version of this article (10.1186/s12917-018-1611-0) contains supplementary material, which is available to authorized users.

## Background

*Staphylococcus aureus* is a leading cause of human nosocomial and community-acquired infections worldwide. Methicillin-resistant *S. aureus* (MRSA) isolates were first identified a few years after the introduction of methicillin in the 1950s following its application in the treatment of penicillin-resistant staphylococcal infections [1, 2]. Since then, MRSA has become recognized as a major health problem in human medicine internationally, especially in hospital settings [3]. Healthcare-associated MRSA (HA-MRSA) causes skin and soft tissue infection like sepsis, septic arthritis, pneumonia etc. [4] whereas community-associated MRSA (CA-MRSA) cause more deadly infection [5] and livestock-associated MRSA (LA-MRSA) are major reservoir of infection [6]. For almost four decades, the increasing prevalence of MRSA strains has posed a major clinical threat to worldwide public health and a significant challenge to the control of infection in human medicine [7].

With the growing prevalence of CA-MRSA [8], the epidemiological aspects of nosocomial infection are also being increasingly studied [9, 10]. Several studies reported that healthy and disease animals usually cow, horses and companion animals can transmit this superbug to human and vice versa [11–14]. Others studies also indicated that companion or pet animals are responsible for household MRSA transmission and may serve as reservoir [6, 15].

Introduced in 1959, methicillin (methicillin, oxacillin, cloxacillin, and flucloxacillin) was the first introduced antimicrobial of the beta-lactam class that is resistant to beta-lactamase inactivation, and it was applied in the treatment of penicillin-resistant *S. aureus.* It is still used as a first-line treatment today, despite the first case of MRSA being reported in England [16] within 2 years of its clinical introduction. MRSA bacteria have developed resistance to all penicillins, including methicillin and other narrow-spectrum β-lactamase penicillin antibiotics [17]. Worldwide, about 2 billion people are carriers of *S. aureus*, and of these, it is likely that 53 million (2.7% of carriers) carry MRSA. Furthermore, an animal-associated clone has been isolated from a human infection [18].

The chromosomally located *mec*A gene encodes the low affinity penicillin-binding protein 2a (PBP-2a) [19] in the presence of high concentrations of β-lactam antibiotics, which can function as a surrogate trans-peptidase capable of ameliorating the four high-affinity PBPs native to *S. aureus* [20]. PBP2 (PBP-2a), constitutively produced in some MRSA isolates; is the main mechanism of resistance [21–23].

In Bangladesh, the prevalence of MRSA in humans has been studied [24] and a recent report provided data on the prevalence of MRSA among dogs and cats in one particular city [25]. In the current study, we determined the prevalence of *S. aureus* isolates including MRSA in a convenience sample collected from hospitals and veterinary centers in selected areas of Bangladesh.

## Methods

### Study period and place

This study was conducted over a 1-year period from September 2013 to August 2014 at the Department of Medicine, Bangladesh Agricultural University (BAU), Mymensingh, Bangladesh, and Mymensingh Medical College, Mymensingh, Bangladesh.

### Sample collection

A total of 100 samples from animals (55 cattle, 36 dogs, and 9 cats) were collected from the BAU Veterinary Clinic, smallholder dairy farms near the BAU campus, and from the Veterinary Hospital in Mymensingh Sadar and the Central Veterinary Hospital in Dhaka. A total of 150 human samples were collected from the Mymensingh Medical College Hospital, Mymensingh, Bangladesh.

Sterilized cotton swab sticks were used to collect samples of pus, mastitic milk, and wound infections. Nasal swabs were collected from normal dogs and cats without any signs of infection. The human sample compositions from hospitalized patients were as follows: surgical wound swabs (*n* = 95), pus from skin infections (*n* = 19), exudates from diabetic ulcers (*n* = 14), exudates from burns swabs (*n* = 13) and aural swabs (n = 9).

### Isolation and identification of *S. aureus*

Bacteria were isolated and identified by their colony morphology, Gram stain results, and biochemical test results (catalase and coagulase tests) according to the report by Quinn and colleagues [26].

### Antimicrobial susceptibility testing

Antimicrobial susceptibility testing was performed using the antimicrobial disc method recommended by the Clinical & Laboratory Standards Institute (CLSI) (www.clsi.org). Seven antimicrobial agents were used to determine the antibiogram of the isolated organisms according to the Gram-positive panel of antimicrobials recommended by the CLSI. The antimicrobial panel comprised: penicillin (10 unit), oxacillin (1 μg), erythromycin (15 μg), ceftazidime (30 μg), gentamicin (10 μg), ciprofloxacin (10 μg), and vancomycin (30 μg).All of the *S. aureus* isolates were tested for their antimicrobial susceptibilities using the Kirby–Bauer disk diffusion technique according to the CLSI 2010 recommendations. All tests were performed using Muller–Hinton agar following 0.5 McFarland standards (1.5 × 10^8^). To standardize the disk potency, a representative disc was tested against the *S. aureus* ATCC 25923 reference strain. The zone of inhibition was compared with the standard value recommended by the CLSI. The results were interpreted as follows: zone of inhibition ≥13 mm = sensitive; zone of inhibition ≤10 mm = resistant.

### Bacterial genomic DNA extraction

The boiling method was used to extract genomic DNA from the isolates [27]. Briefly, a single *S. aureus* colony was inoculated into 100 μl of distilled water in an eppendorf tube, mixed well, and then boiled for 10 min. After boiling, the tubes were immediately put on ice and then centrifuged at 9000×*g* for 10 min at 4 °C. The bacterial DNA-containing supernatant was collected and used as a DNA template for multiplex PCR.

### Amplification of genus- and species-specific *S. aureus* genes

Methicillin-resistant staphylococci were identified by PCR amplification of the *mec*A gene. DNA was extracted from *S. aureus* cultures and amplified with primers for the targeted *Staphylococcus* genus-specific 16S rRNA gene, the staphylococcus species-specific *nuc* gene, and the MRSA-specific *mec*A gene.

PCRs were performed in a gradient thermal cycler (Eppendorf, Hamburg, Germany). The *S. aureus*-specific *nuc* gene (279 bp), methicillin resistance *mec*A gene (147 bp), and *Staphylococcus* genus-specific 16S rRNA gene (756 bp) were detected. Previously reported primers were used, along with *Staphylococcus* genus-specific 16S rRNA (756 bp) as an internal control [28]. Each 25-μl reaction mixture contained 5μlof genomic DNA, 12.5 μl of PCR master mix (Promega Corporation, Madison, WI, USA), 1 μl of 100 pmol of the forward and reverse primers, and the final volume was adjusted to 25 μl with 5.5 μl of nuclease-free water. DNA amplification involved denaturation at 94 °C for 1 min, followed by 30 cycles at 94 °C for 30 s, 55 °C for 30 s, and 72 °C for 1 min, with a final elongation step at 72 °C for 5 min. The PCR products were analyzed by 1% agarose gel electrophoresis (Alpha Imager, Wiesbaden Germany), with ethidium bromide staining, and a gel documentation system (Alpha Imager) was used for photography.

### Documentation and visualization of DNA samples

Following electrophoresis, PCR products were visualized using a UV transilluminator. Bands of 157 bp (*mec*A), 297 bp (*nuc*), and 756 bp (16S rRNA) indicated positive results.

## Results

A total of 100 samples from animals (cattle: 55, dogs: 36, cats: 9) (Table 1) and 150 samples from humans were included in this study (Table 2). Of these100 samples of animal origin, 29 were positive for *S. aureus* among the different animals*.* Among these 29 samples, four from dogs were MRSA-positive, while those from cattle and cats were MRSA-negative.

**Table 1:** Detection of *S. aureus* and MRSA among specimens from different animals sampled from September, 2013 to August, 2014

Animal	No. tested	*S. aureus* positive (%)	MRSA positive (%)
Cattle (Mastitic milk, pus and wound swab)	55	18 (32.7)	–
Dog (Nasal Swab)	36	09 (25.0)	04 (44.4)
Cat (Nasal Swab)	09	02 (22.2)	–
Total	100	29 (29.0)	04 (13.8)

49. -: Not detected

**Table 2:** Detection of *S. aureus* and MRSA among various specimens from humans sampled from September, 2013 to August, 2014

Types of specimens	No. tested	*S. aureus* positive (%)	MRSA positive (%)
Surgical wound swab	95	34 (35.8)	18 (52.9)
Pus from skin infection	19	11 (57.9)	7 (63.6)
Exudates from diabetic ulcer	14	07 (50.0)	4 (57.1)
Exudates from burn	13	07 (53.8)	3 (42.9)
Aural swab	9	05 (55.6)	2 (40.0)
Total	150	64 (42.7)	34 (53.1)

**Fig. 1:**
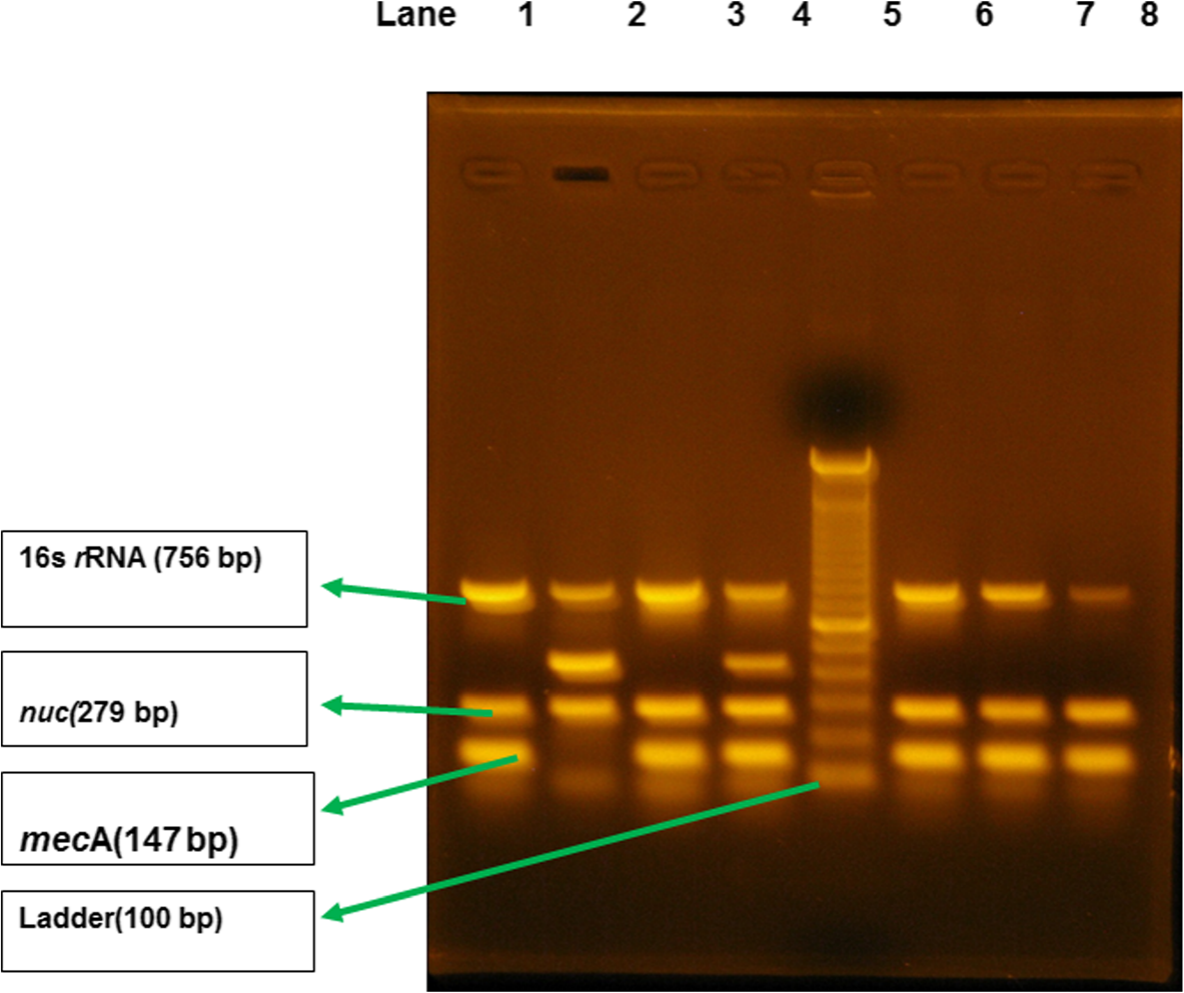
Multiplex PCR assay to identify *mec*A (157 bp), *nuc* (297 bp), and 16S rRNA genes (756 bp) in *S. aureus*

The samples from humans were collected from hospitalized patients. Of the 150 samples, 64 were *S. aureus*-positive*.* Among these 64, 34 were found to be positive for MRSA of human origin (Table 2). The growth characteristics, staining, and coagulase test results were all consistent with *S. aureus*. We confirmed the presence of the *mec*A (147 bp), *nuc* (279 bp), and 16S rRNA genes by multiplex PCR (Fig. 1).

Antimicrobial susceptibility testing of the isolated organisms with an antimicrobial panel was performed by the disk diffusion method using the Kirby–Bauer technique according to the CLSI 2010 guidelines. All of the tests were performed on Muller–Hinton agar. Seven antimicrobial agents were used to determine the antibiogram of the isolated organisms according to the Gram-positive panel of antimicrobials (penicillin, gentamicin, oxacillin, erythromycin, vancomycin, ciprofloxacin, ceftazidime) recommended by the CLSI. Plates were incubated at 35 °C for 24 h, after which the inhibition zone was measured. The patterns of antimicrobial resistance among the isolates typed as MRSA are shown in Table 3. All 34 isolates were resistant to penicillin and oxacillin, and susceptible to vancomycin.

**Table 3:** Frequency of drug resistance in MRSA from animals and humans from September 2013 to August, 2014

Antimicrobials	Humans		Animal	
	No. of MRSA isolates (*n* = 34)	% of resistant isolates	No. of MRSA isolates (*n* = 4)	% of resistant isolates
Penicillin	34	100	–	–
Oxacillin	34	100	4	100
Erythromycin	25	73	–	–
Ciprofloxacin	24	70	–	–
Gentamicin	23	67	–	–
Ceftazidime	21	61	–	–
Vancomycin	–	–	–	–

-: Not detected

## Discussion

After the introduction of β-lactam antimicrobials, the prevalence of MRSA infections and colonization increased steadily over time [27, 29]. Careful monitoring of the occurrence of MRSA is important for appropriate clinical management of hospital patients and for studying MRSA epidemiology in the community. In the present study, the prevalence of staphylococci (*S. aureus*) was 25% among dogs, which was lower than two previous studies, 91% [30] and 65.1% [31], among dogs in a referral animal hospital in the UK. However, those studies used samples from a limited number of hospitalized dogs and more than one sampling site. By contrast, the present study investigated the prevalence of MRSA in a larger vet-visiting dog community and omitted hospitalized dogs, which may be more representative of the healthy dog population in Bangladesh. Few studies have reported the overall prevalence of *S. aureus*, so it is not possible to draw comparisons between different populations and countries. The present study showed that staphylococci carriage is not unusual in the nasal mucosa of dogs in Bangladesh. Although the proportion of isolated staphylococci was low in our study, the prevalence of MRSA was 11.1% among the dog population studied. Another recent study reported MRSA among dogs in Bangladesh [25], specifically MRSA was detected from nasal swabs, which was consistent with our study; however, their report was limited to a small confined area and included healthy and diseased dogs. Three other previous studies reported MRSA detection rates of 5%, 8%, and 21.4% among the dog population in Jordan, Finland, and France, respectively [32–34]. Most importantly, other studies have also reported that canine MRSA strains reflect those prevalent in human hospital settings [35–37]. However, wounds are the major site of infection for MRSA in dogs, cats, and horses [38, 39], but all of the MRSA-positive samples were from nasal swabs. It is unclear to what extent MRSA carried in dogs is a potential source of transmission to humans and vice versa. MRSA among dairy cattle has been reported elsewhere [6, 12]. The classes of antimicrobials commonly used in Bangladesh include penicillins, fluroquinolones, sulfonamides, tetracyclines, and aminoglycosides. Among these, tetracyclines are the most frequently used class of antimicrobials [40] which might be one of the causes why no MRSA was found in cattle. Our investigation revealed an overall prevalence of MRSA of 53.1% for the *S. aureus* samples from humans which was slightly higher than Khan and coworker’s study [24] in Bangladesh. The prevalence of human MRSA in our study was also similar to that reported in studies from more developed countries such as Japan (52%) [41] and the USA (54%) [42]. There was no correlation between our study findings and those of a previous Bangladeshi study by Jinnah and colleagues where the occurrence of MRSA was reported to be 21.6% [43]. The occurrence of MRSA for humans in our study was also dissimilar to that of an Indian study (8%) [44].

A significant increase in the prevalence of MRSA among the Bangladeshi population was reported in a previous study [45], which noted an increase in prevalence over time. This may be due to the widespread, excessive use of antimicrobials over recent years. The aim of the present study was to determine the rate of MRSA-positivity among isolates from patients and animals. We have shown that *nuc* and *mec*A gene amplification by multiplex PCR as an efficient and rapid method to detect and identify MRSA from cultured specimens. Adopting this method may provide substantial benefits for infection control by allowing for precise and cost-effective control measures to be implemented. Our results showed that MRSA carriage among humans and animals threatens the effective antimicrobial treatment of infections with this bacterium, and the widespread use of antimicrobials may increase the risk of resistance gradually arising [46]. Consistent with our findings, macrolide and ciprofloxacin resistance was previously reported in MRSA [47, 48]. Further studies on the distribution and persistence of MRSA strain reservoirs among animals and humans along with specific resistance patterns are now required.

Our study had some limitations that are worth noting. Domestic animals were only tested in urban areas, and many pet owners were reluctant to provide samples from their animals. This bacterium has public health implications for the owners since they might be infected with the same strains. As a result, it was difficult to obtain a representative number of samples to analyze. In future studies, further molecular characterization is needed to investigate the transmission of MRSA from animal species to humans by analyzing the genetic relatedness of the prevalent strains in humans and domestic animals in Bangladesh.

## Conclusions

We detected *S. aureus* and MRSA in both humans and dogs. Some isolates were found to be resistant to antimicrobials available locally in Bangladesh. MRSA carriage in humans and animals appears to be a great threat to effective antimicrobial treatment. Therefore, our findings will encourage clinicians and health care institutions to adopt precise guidelines about the use of antimicrobials regarding MRSA patient treatment. Further studies are required to better understand the epidemiology of MRSA human–animal inter-species transmission.

## Additional files

Additional file 1: Data on clinical samples from humans used for the detection of *Staphylococcus aureus* and MRSA in Bangladesh. (CSV 7 kb)

Additional file 2: Data on clinical samples from animals used for the detection of *Staphylococcus aureus* and MRSA in Bangladesh. (CSV 11 kb)

## Abbreviations

CLSI: Clinical and Laboratory Standards Institute
MRSA: Methicillin-resistant *Staphylococcus aureus*
PCR: Polymerase chain reaction
